# Characterization of the T‐cell response to Dau c 1, the Bet v 1‐homolog in carrot

**DOI:** 10.1111/all.12938

**Published:** 2016-06-10

**Authors:** N. Zulehner, B. Nagl, P. Briza, A. Roulias, B. Ballmer‐Weber, G. J. Zlabinger, F. Ferreira, B. Bohle

**Affiliations:** ^1^ Department of Pathophysiology and Allergy Research and Christian Doppler Laboratory for ImmunomodulationMedical University of ViennaViennaAustria; ^2^ Department of Molecular BiologyUniversity of SalzburgSalzburgAustria; ^3^ Allergy UnitDepartment of DermatologyUniversity Hospital ZurichZurichSwitzerland; ^4^ Institute of ImmunologyMedical University of ViennaViennaAustria

**Keywords:** allergic sensitization, Bet v 1, birch pollen‐associated food allergy, Dau c 1, T cells

## Abstract

**Background:**

In contrast to other Bet v 1‐related food allergens, the major carrot allergen, Dau c 1, has been suggested to induce food allergy independently from Bet v 1. As T cells are crucial in the sensitization process, we sought to characterize the T‐cell response to Dau c 1 and its cross‐reactivity with Bet v 1.

**Methods:**

Dau c 1‐specific T‐cell lines (TCL) and clones (TCC) established from PBMC of birch pollen‐allergic patients with carrot allergy were analyzed for reactivity to Bet v 1, epitope specificity, allergen‐induced cytokine secretion, and expression of integrins α4β7 and α4β1, critical for gut and lung homing, respectively. mRNA expression of GATA3 and Tbet was analyzed in sorted CD3^+^ CD4^+^ CFSE ^low^ cells proliferating upon stimulation of PBMC with Dau c 1 or Bet v 1. Dau c 1 was incubated with endolysosomal proteases, and the resulting fragments were identified by mass spectrometry.

**Results:**

Among 14 distinct T‐cell‐activating regions, Dau c 1_139–153_ was recognized by 55% of the patients. Only 6 of 15 (40%) Dau c 1‐specific TCL and 9 of 21 (43%) TCC reacted with Bet v 1. Bet v 1‐nonreactive TCC were mainly Th1‐like and showed a higher expression of the integrin β7 and a significantly lower expression of the integrin β1 than Bet v 1‐positive TCC. A Th1‐like response was also detected in Dau c 1‐reactive CD3^+^ CD4^+^ CFSE ^low^ cells. Full‐length Dau c 1 was still detectable after 48 h of endolysosomal degradation. Proteolytic fragments of Dau c 1 matched its T‐cell‐activating regions.

**Conclusion:**

Dau c 1 displays several characteristics of sensitizing allergens, namely a major T‐cell‐activating region, low susceptibility to endolysosomal degradation, and induction of a Bet v 1‐independent T‐cell response. These cellular insights confirm that the major carrot allergen has a special status among Bet v 1‐related food allergens.

**Abbreviations**
aa: amino acid
APC: antigen‐presenting cells
BPE: birch pollen extract
cpm: counts per minute
MFI: mean fluorescence intensity
PBMC: peripheral blood mononuclear cells
SI: stimulation index
TCC: T‐cell clone
TCL: T‐cell line

Only recently, the prevalence of sensitization to carrot in 13.000 German children aged 3–17 years was shown to be 8% 1. Carrot allergy is generally considered a birch pollen‐related food allergy with Dau c 1 as major allergen 2. Birch pollen‐related food allergy results from primary sensitization to the major birch pollen allergen followed by cross‐reactivity of Bet v 1‐specific IgE antibodies and T cells with Bet v 1‐related food proteins 3, 4, 5, 6. Consequently, Bet v 1 and homologous food allergens are usually not considered to show sensitizing activity. Dau c 1 possesses an amino acid (aa) sequence similarity of 61% (38% identity) with Bet v 1 and shows a similar 3‐dimensional structure 7, 8. However, comparisons of the surface topology and physicochemical properties of Dau c 1 and Bet v 1 have revealed that the two proteins share only some but not all IgE epitopes 8. Indeed, IgE binding to Dau c 1 could not be inhibited by Bet v 1 in a subset of birch pollen‐allergic patients with carrot allergy 9. This observation clearly differed from other Bet v 1‐related food allergens, in particular from Api g 1 in celery, which has an aa sequence similarity of 93% (81% identity) with Dau c 1 7. In contrast to Dau c 1, IgE binding to Api g 1 is completely abolished by Bet v 1 10, 11. Additional evidence for a Bet v 1‐independent Dau c 1‐specific IgE response has been provided by the detection of significant Dau c 1‐specific IgE levels in 4 adult patients with strong immediate systemic reactions to raw carrot who displayed no Bet v 1‐specific IgE antibodies 12. The same applied to two patients with carrot‐induced asthma 13. Together, these data provided evidence that Dau c 1 might initiate food allergy independently from the major birch pollen allergen making it an atypical Bet v 1‐related food allergen.

Allergen‐specific CD4^+^ T cells are crucial in the sensitization process of IgE‐mediated allergy as clonal expansion of allergen‐specific Th2 cells that produce IL‐4 and IL‐13 promotes B‐cell class switching to the production of allergen‐specific IgE antibodies. As information on the T‐cell response to the carrot allergen is scarce, we characterized Dau c 1‐specific T‐cell lines (TCL) and clones (TCC) expanded from PBMC of Bet v 1‐ and Dau c 1‐sensitized birch pollen‐allergic patients with carrot allergy regarding their phenotype and cross‐reactivity with the major birch pollen allergen. To gain insight into the possible priming sites of Dau c 1‐specific T cells, we compared the expression of the integrins β7 and β1 on the surface of Dau c 1‐specific TCC cross‐reactive and nonreactive with Bet v 1. The gut‐homing factor integrin α4β7 binds to the key intestinal mucosal addressin cell adhesion molecule‐1 (MAdCAM‐1) 14 and has been shown to be expressed on milk allergen‐specific T cells and peanut‐specific TCC 15, 16. In contrast, the α4β1‐integrin (very late antigen‐4) has been implicated in the recruitment of T cells to extraintestinal sites of inflammation, such as lungs and skin 17. We also determined T‐cell‐activating regions of Dau c 1 and subjected the protein to endolysosomal degradation as stability to lysosomal proteolysis has been considered a relevant factor for immunogenicity 18, 19. The resulting proteolytic fragments were sequenced by mass spectrometry and compared with the identified T‐cell‐activating regions.

## Methods

### Allergens and peptides

Recombinant Dau c 1, Bet v 1, Api g 1, and Mal d 1 were purchased from Biomay (Vienna, Austria). Recombinant Cor a 1 was provided by S. Scheurer (Paul Ehrlich Institute, Langen, Germany). A panel of 48 synthetic 12‐mer peptides overlapping by 9 aa and representing the entire aa sequence of Dau c 1 was purchased from Thermo Fisher Scientific (Uppsala, Sweden).

### Allergic patients

We included 31 patients with birch pollen allergy proven by typical case history and birch pollen‐specific IgE levels of >0.35 kU_A_/L (ImmunoCAP; Thermo Fisher Scientific). All patients reported allergic symptoms after ingestion of raw carrot. In addition, patients 1, 5, 10, 13, and 20 had been positive in double‐blind placebo‐controlled food challenges with carrot 9. All patients displayed Dau c 1‐specific IgE as determined by ELISA (data not shown). The study was approved by the local ethics committees and all donors provided written informed consent.

### Dau c 1‐specific T‐cell lines (TCL)

PBMC (1.5 × 10^6^) were stimulated with Dau c 1 (5 μg/ml). Cultures without Dau c 1 served as controls. After 4 days, human rIL‐2 (10 U/ml; Roche, Basel, Switzerland) was added. After another 4 days, viable T‐cell blasts were enriched by density centrifugation and subjected to the cloning procedure. Remaining blasts were expanded with rIL‐2 (10 U/ml, Boehringer Mannheim, Mannheim, Germany) and irradiated PBMC (1 × 10^5^) in 200 μl UltraCulture Medium (BioWhittaker, Walkersville, MD, USA) supplemented with 2 mM glutamine, 2 × 10^−5^ M 2‐mercaptoethanol, and gentamicin (Lonza, Basel, Switzerland). After sufficient cell numbers were obtained, cells were rested for 14 days and stimulated with recombinant allergens (each 5 μg/ml) or synthetic 12mer peptides (5 μg/ml) in the presence of irradiated autologous PBMC. After 48 h, proliferative responses were determined by ^3^H‐thymidine incorporation assay. The stimulation index (SI) was calculated as ratio between counts per minute (cpm) obtained in cultures with TCL plus autologous PBMC plus allergen/peptide and cpm obtained in cultures containing only TCL plus autologous PBMC.

### Dau c 1‐specific T‐cell clones

T‐cell blasts from Dau c 1‐specific TCL were seeded by means of limiting dilution technique in 96‐well round‐bottomed plates (Nunclon; Nunc, Copenhagen, Denmark) in the presence of irradiated PBMC/0.2% v/v PHA (GE Healthcare, Little Chalfont, UK) and rIL‐2 (4 U/well) in the medium described above. Grown clones were screened for allergen specificity by incubation with Dau c 1 (5 μg/ml) in the presence of irradiated autologous PBMC. TCC in medium alone and PBMC without TCC served as controls. The supernatants of clones that reacted to Dau c 1 (SI > 10) were harvested after 24 h, and cytokine levels were determined using the Luminex System 100 (Luminex, Austin, TX, USA). A ratio of IFN‐γ/IL‐4 > 5 was classified as Th1‐like, 0.2–5 as Th0, and <0.2 as Th2‐like. These clones were expanded by alternating turns of stimulation with irradiated PBMC plus either allergen or IL‐2 and as soon as possible tested for cross‐reactivity and epitope specificity as described above for TCL. In addition, TCC were stained with CD3‐APC (BD Bioscience, Franklin Lakes, NJ, USA), CD4‐PerCP (eBioscience, San Diego, CA, USA), TCRαβ‐FITC and integrin β7‐PE (BD Pharmingen, San Jose, CA, USA), and integrin β1‐FITC (Beckman Coulter, Brea, CA, USA) or isotype‐matched antibodies as negative controls.

### Detection of GATA3 and Tbet expression

PBMC were stained with 0.5 μM CFSE (Invitrogen, Camarillo, CA, USA) and stimulated with either Bet v 1 or Dau c 1 (10 μg/ml) in AIM‐V medium (Invitrogen). After 8 days, CD3^+^CD4^+^CFSE^low^ cells were sorted using a MoFlo Astrios (Beckman Coulter) cell sorter. RNA of sorted cells was isolated using RNeasy Mini Kit (Qiagen, Hamburg, Germany) and reversely transcribed with High Capacity cDNA Reverse Transcription Kit (Applied Biosystems, Foster City, CA, USA) according to the manufacturer's protocol. Quantitative real‐time PCR was performed with Power SYBR Green Master Mix (Applied Biosystems) and published primers for EF‐1α (endogenous control), GATA3, and Tbet 20 in an ABI 7900 HT Sequence Detection System (Applied Biosystems). All amplifications were performed in triplicate. Relative quantification was performed using DataAssist Software v2.0 (Applied Biosystems).

### Endolysosomal degradation assay

Dau c 1 (5 μg) and Api g 1 (5 μg) were incubated with microsomal enzyme extracts (7 μg) isolated from monocyte‐derived dendritic cells as described previously 21. Proteolysis was stopped after 1, 3, 6, 12, 24, 36, or 48 h by heat denaturation, and samples were analyzed by SDS‐PAGE (15%) and mass spectrometry using a Q‐Exactive mass spectrometer (Thermo Fisher Scientific) coupled to a capillary rpHPLC (Dionex, Germering, Germany). Peptides were identified using PEAKS Studio 7.0 (Bioinformatics Solutions, Waterloo, ON, Canada).

## Results

### T‐cell epitopes of Dau c 1

**Figure 1:**
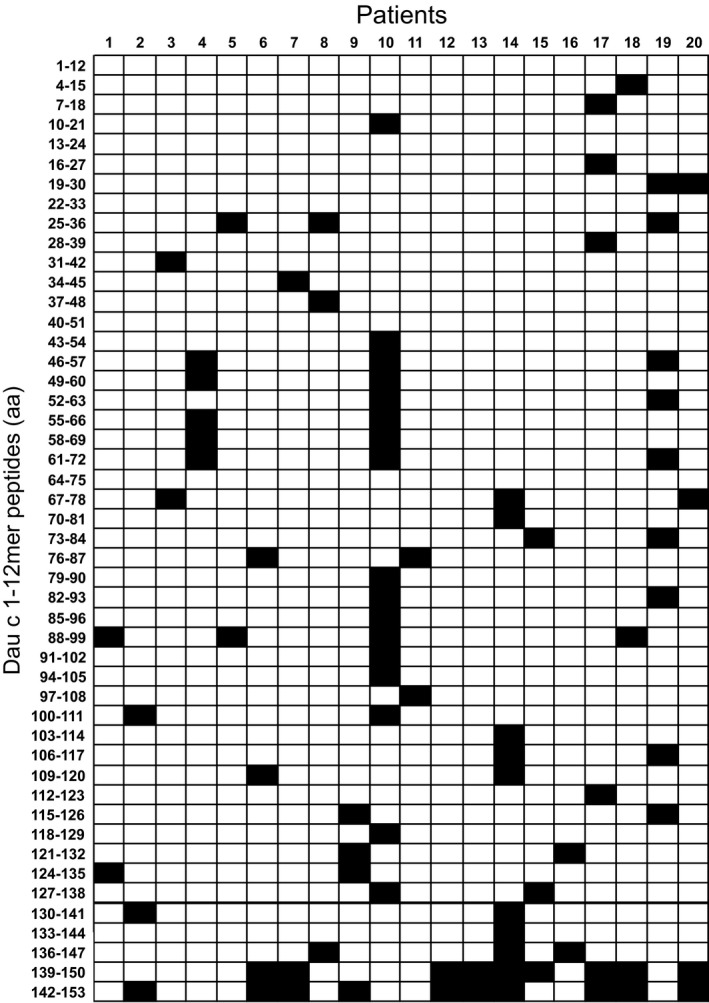
T‐cell‐activating regions of Dau c 1. Proliferation of Dau c 1‐specific TCL from 20 different patients in response to 48 overlapping peptides spanning the aa sequence of Dau c 1 is shown. Peptides inducing SI > 2 are denoted in black.

In total, Dau c 1‐specific TCL from 20 different patients could be expanded and subjected to epitope mapping experiments. Proliferative responses with SI > 2 were considered positive (Fig. 1). Several peptides within the regions Dau c 1_4–21_, Dau c 1_16–30_, Dau c 1_28–48_, Dau c 1_43–54_, Dau c 1_49–69_, Dau c 1_70–96_, and Dau c 1_91–144_ were recognized by one or two patients. Three patients (15%) harbored T cells reacting to peptides located at Dau c 1_25–36_, Dau c 1_46–57_, Dau c 1_61–72_, Dau c 1_67–78_, and Dau c 1_136–147_. Dau c 1_88–99_ was recognized by four patients (20%). Eleven of 20 (55%) Dau c 1‐specific TCL showed proliferative responses to peptides covering the region Dau c 1_139–153_. The peptides covering aa 1–12, 13–24, 22–33, 40–51, and 64–75 did not induce proliferation in any of the Dau c 1‐specific TCL.

### Cross‐reactivity of Dau c 1‐specific T‐cell lines with Bet v 1 and homologous food allergens

Fifteen Dau c 1‐specific TCL were additionally stimulated with Bet v 1, Api g 1 (celery), Mal d 1 (apple), and Cor a 1 (hazelnut), and proliferative responses were determined (Table 1). Considering SI > 2 positive, 6 of 15 (40%) Dau c 1‐specific TCL cross‐reacted with Bet v 1. Moreover, 7 of 14 (50%) Dau c 1‐specific TCL reacted to Api g 1, 5 of 11 (45%) recognized Mal d 1, and 7 of 15 TCL (47%) cross‐reacted with Cor a 1 (Table 1).

**Table 1:** Reactivity of Dau c 1‐specific TCL with Bet v 1 and homologous food allergens

Pat. no.	1*	2	3	6	7	8	9	10	12	13	15	17	18	19	20
Dau c 1	**2.4** **	**6.3**	**2.6**	**12.9**	**3.8**	**11.6**	**107**	**2.1**	**3.0**	**2.1**	**6.3**	**4.4**	**5.5**	**6.1**	**29.3**
Bet v 1	1.0	0.9	1.0	1.1	1.0	**3.5**	**87.3**	**2.5**	**2.2**	0.9	1.4	**2.5**	1.7	1.4	**4.2**
Api g 1	0.9	1.9	1.1	**14.8**	1.7	**3.1**	**13.4**	1.4	**2.8**	0.8	n.d.	**2.7**	**3.9**	1.0	**19.4**
Mal d 1	n.d.	0.8	1.0	0.5	0.7	**13.2**	**64.8**	n.d.	0.5	n.d.	n.d.	1.4	**2.8**	**3.5**	**8.2**
Cor a 1	1.2	1.7	1.5	**10.3**	**2.7**	0.9	**6.9**	1.1	1.4	1.3	**2.2**	**2.3**	**3.9**	0.8	**3.1**

*Same patient numbering as in Fig. 1, **positive responses are shown in bold; n.d. not determined.

### Characterization of Dau c 1‐specific T‐cell clones

In total, 22 CD3^+^CD4^+^TCRαβ^+^ Dau c 1‐specific TCC were expanded from 15 different donors (Table 2). Twenty‐one TCC could be tested for cross‐reactivity with Bet v 1 and 9 (43%) were positive. Five of these TCC had lost their reactivity to Dau c 1 but still responded strongly to Bet v 1. Similarly, we have previously observed that TCC expanded with Mal d 1 and Api g 1 became nonreactive to the food allergens during the *in vitro* expansion process but remained reactive with Bet v 1 4, 11. Analysis of the levels of IL‐4, IFN‐γ, and IL‐10 in response to stimulation with Dau c 1 revealed that 6 clones (27%) belonged to the Th2 subset, 10 TCC (45%) were Th1‐like, and six clones (27%) were classified as Th0 clones (Table 2). Thirteen TCC (59%) synthesized IL‐10 (>50 pg/ml). Six Dau c 1‐specific TCC could be expanded to cell numbers sufficient for epitope mapping (Table 2). Three clones (50%) recognized the C‐terminal region Dau c 1_139–153_, two TCC reacted to the peptide Dau c 1_34–48_, and one TCC reacted to Dau c 1_67–78_.

**Table 2:** Characterization of Dau c 1‐specific TCC

	TCC	Stimuli		Cytokines [pg/ml]			Subset	Peptide	
		Dau c 1	Bet v 1	IL‐4	IFN‐γ	IL‐10		aa no.	Sequence
1	P1 36	1.1	**35.4** *	198	500	180	Th0	n.d.	
2	P2 137	1.0	**181**	406	<9.5	370	Th2	34–48	APGAYKSVEVKGDGG
3	P3 5	1.6	**91**	425	<9.5	502	Th2	n.d.	
4	P3 13	**112**	2.1	230	<9.5	260	Th2	139–153	NTALFKAIEAYLIAN
5	P3 56	0.8	**36.2**	27.0	<9.5	63.1	Th0	n.d.	
6	P3 66	0.9	**5.1**	<0.9	510	33.2	Th1	n.d.	
7	P4 17	**2.0**	**2.6**	<0.9	173	<7.5	Th1	n.d.	
8	P4 29	**2.5**	**2.4**	<0.9	<9.5	<7.5	Th0	n.d.	
9	P5 2	**2.1**	**2.0**	<0.9	21.1	<7.5	Th0	n.d.	
10	P6 2	**13.0**	0.5	339	<9.5	15.8	Th2	142–153	LFKAIEAYLIAN
11	P6 3	**8.9**	**2.7**	<0.9	<9.5	<7.5	Th0	n.d.	
12	P7 54	**3.9**	0.6	<0.9	<9.5	692	Th0	34–48	APGAYKSVEVKGDGG
13	P8 117	**15.4**	1.1	17.2	2319	118	Th1	67–78	TVRTDAVNKEAL
14	P9 16	**40.4**	1.0	<0.9	502	100	Th1	n.d.	
15	P10 41	**4.7**	1.1	<0.9	102	17.1	Th1	n.d.	
16	P12 36	**2.3**	0.8	12 296	536	81.7	Th2	n.d.	
17	P12 84	**5.2**	0.7	213	1377	32.3	Th1	n.d.	
18	P13 52	**7.4**	1.8	<0.9	172	20.1	Th1	n.d.	
19	P14 145	**48.1**	1.0	<0.9	424	190	Th1	n.d.	
20	P14 149	**28.4**	0.6	423	<9.5	60.5	Th2	139–153	NTALFKAIEAYLIAN
21	P15 55	**50.0**	0.8	<0.9	523	112	Th1	n.d.	
22	P17 62	**10.5**	n.d.	<0.9	1946	<7.5	Th1	n.d.	

*Positive responses are shown in bold, n.d. not determined.

Ten Dau c 1‐specific TCC from 6 different patients could be analyzed for the expression of integrins β7 and β1 (Table 3). As observed previously, TCC constitutively expressed both integrins independently from their activation status 16. However, Bet v 1‐nonreactive TCC showed a slightly higher surface expression of integrin β7 and a significantly lower expression of integrin β1 (CD29, *P* = 0.032, Mann–Whitney *U*‐test) than Bet v 1‐cross‐reactive Dau c 1‐specific TCC.

**Table 3:** Integrin expression of Bet v 1‐reactive and nonreactive Dau c 1‐specific TCC

TCC	Proliferation (SI) to		Subset	Expression (MFI) of		TCC	Proliferation (SI) to		Subset	Expression (MFI) of	
	Dau c 1	Bet v 1		Integrin β7	Integrin β1		Dau c 1	Bet v 1		Integrin β7	Integrin β1
P1 36	1.1	35.4	Th0	547	839	P3 13	112	2.1	Th2	201	174
P2 137	1.0	181	Th2	184	1564	P9 16	40.4	1.0	Th1	412	541
P3 5	1.6	91	Th2	280	1341	P10 41	4.7	1.1	Th1	1075	636
P3 56	0.8	36.2	Th0	554	851	P14 137	207	1.1	Th1	886	330
P3 66	0.9	5.1	Th1	1059	1666	P14 145	48.1	1.0	Th1	1038	974
Median				547	1341*	Median				886	541*

MFI, mean fluorescence intensity; *P < 0.05, Mann–Whitney *U*‐test.

### Th subsets in Dau c 1^+^Bet v 1^+^ and Dau c 1^+^Bet v 1^−^ TCC

By comparing the subset distribution of Bet v 1‐reactive and nonreactive Dau c 1‐specific TCC, we found that 7 of 12 (58%) Bet v 1^−^ and 2 of 9 (22%) Bet v 1^+^ clones were Th1‐like (Table 2). Furthermore, 8 of 12 (67%) Bet v 1^−^ clones produced IL‐10 in contrast to 4 of 9 (44%) Bet v 1^+^ TCC. These data pointed to a difference in effector function between Bet v 1‐cross‐reactive and nonreactive TCC.

**Figure 2:**
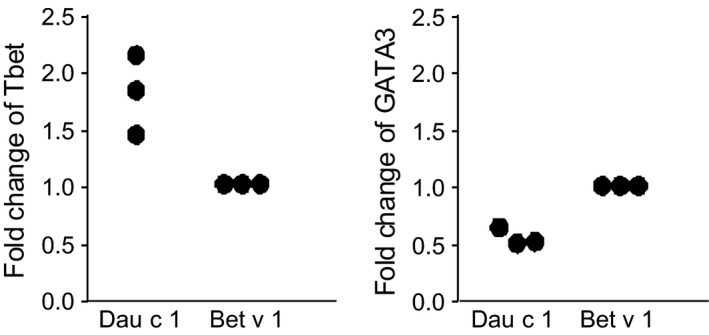
Dau c 1‐reactive T cells are Th1‐like. CFSE‐stained PBMC of three allergic patients were stimulated once with Dau c 1 or Bet v 1. After 8 days, proliferated CD3^+^CD4^+^CFSE^low^ cells were sorted and analyzed for mRNA expression of Tbet or GATA3 by qPCR. Fold changes of Dau c 1‐stimulated T cells to normalized values of Bet v 1‐stimulated T cells are shown.

To confirm the discrepancy in cytokine production of Dau c 1‐ and Bet v 1‐reactive clones in specific polyclonal T cells, we analyzed the mRNA expression of the major transcription factors for Th2 (GATA3) and Th1 (Tbet) in CD4^+^CD3^+^ cells that proliferated upon a single stimulation of PBMC with either allergen. However, due to the very low frequency of allergen‐specific T cells in the periphery, sufficient numbers of proliferating CD4^+^ T cells could be sorted from solely three individuals (Fig. 2). Nevertheless, a higher expression of Tbet was detected in Dau c 1‐reactive CFSE^low^CD4^+^CD3^+^ cells when compared to CFSE^low^CD4^+^CD3^+^ cells in cultures stimulated with Bet v 1. *Vice versa*, Bet v 1‐reactive T cells expressed higher levels of GATA3 (Fig. 2).

### Endolysosomal degradation of Dau c 1

**Figure 3:**
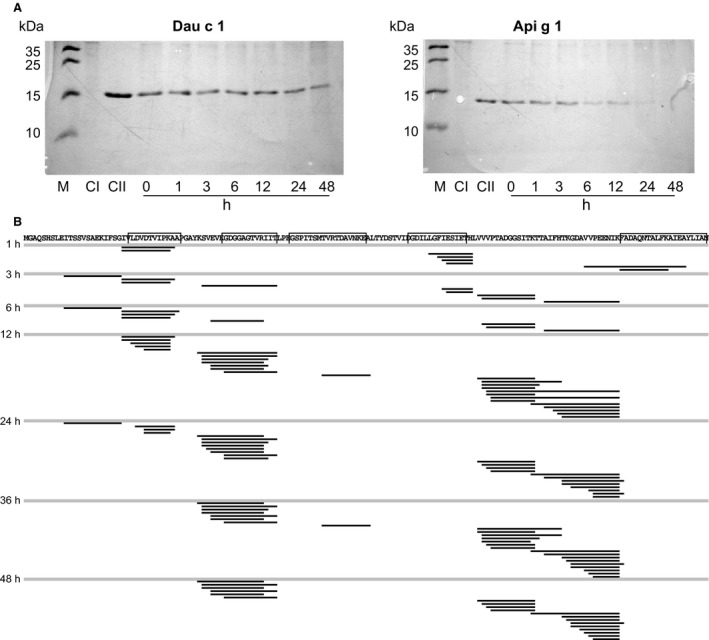
Endolysosomal degradation of Dau c 1. (A) Dau c 1 and Api g 1 were incubated with endolysosomal proteases for indicated time periods and analyzed by SDS‐PAGE (15%) and Coomassie staining. M, marker proteins; CI, proteases without allergen; CII, allergen without proteases; (B). Dau c 1‐derived peptide sequences after endolysosomal proteolysis for 1, 3, 6, 12, 24, 36, and 48 h. Each line represents a unique peptide found. T‐cell‐activating regions recognized by >15% of the patients are framed in the aa sequence of Dau c 1.

The characterization of the T‐cell response to Dau c 1 indicated that the carrot allergen differed from other Bet v 1‐related food allergens, in particular from the highly homologous celery allergen Api g 1. Api g 1 lacks a major T‐cell‐activating region and displays strong cellular cross‐reactivity with Bet v 1 11. To check whether this divergence resulted from differences in antigen processing, we compared the endolysosomal proteolysis of both proteins (Fig. 3A). Api g 1 was degraded faster and only detectable for 12 h, whereas full‐length Dau c 1 was still detectable at 48 h. We also sequenced the proteolytic fragments of Dau c 1 after 1, 3, 6, 12, 24, and 48 h (Fig. 3B). Peptide clusters within the regions Dau c 1_23–34_, Dau c 1_92–101_, and Dau c 1_127–149_ were detected after 1 h of incubation. After 3 h, additional clusters within Dau c 1_10–22_, Dau c 1_41–57_, Dau c 1_103–115_, and Dau c 1_118–134_ were identified and complemented by Dau c 1_68–78_ after 12 h. Overall, the proteolytic peptide clusters matched the T‐cell‐activating regions identified in Dau c 1‐specific TCL (Fig. 1). The most frequently recognized region Dau c 1_139–153_ appeared after 1 h of endolysosomal degradation.

## Discussion

In the past, we and others have characterized the T‐cell response to Api g 1, Cor a 1, and Mal d 1, and their cellular cross‐reactivity with the major birch pollen allergen 4, 11, 21, 22, 23, 24. These studies have shown that T lymphocytes reactive with these food allergens are mainly cross‐reactive Bet v 1‐specific T cells. Here, we applied the same approach to study Dau c 1‐specific T cells and provide strong evidence that the major carrot allergen may induce T‐cell responses independently from Bet v 1. These findings complement previous findings on Bet v 1‐independent IgE responses to Dau c 1 in carrot‐allergic individuals 9, 12, 13.

Most surprisingly, the T‐cell response to Dau c 1 differed notably from Api g 1 although both proteins have 93% aa sequence similarity 7. Api g 1 is devoid of immunodominant T‐cell‐activating regions recognized by more than 50% of birch pollen‐allergic individuals with celery allergy 11. In contrast, the region Dau c 1_139–153_ was recognized by 55% of the patients in this study (Fig. 1). The same region had already previously attracted our attention as the majority of Cor a 1‐specific TCC reactive to epitopes located within aa 142–153 cross‐reacted with Dau c 1 but not with Bet v 1 22. In the present study, all seven Cor a 1‐reactive Dau c 1‐specific TCL harbored T cells specific for epitopes within aa 142–153 and four of them did not react to Bet v 1. These findings demonstrate the existence of food‐specific T‐cell epitopes in the immunodominant region Dau c 1_142–153_ that are shared with the major allergen in hazelnut but not with Bet v 1.

We recently have found that the sensitizing activity of Bet v 1‐related food allergens correlates with the presence of immunodominant T‐cell‐activating regions 21. The nonsensitizing proteins Api g 1 and Mal d 1 lack major T‐cell epitopes, whereas the major sensitizer Bet v 1 contains the region Bet v 1_142–156_ recognized by 63% of birch pollen‐allergic patients. Dau c 1 confirms these findings as it contains an immunodominant T‐cell‐activating region and induces IgE responses independently from Bet v 1 9, 12, 13. Beyond that, a high proportion of Dau c 1 remained intact for 48 h of incubation with endolysosomal proteases (Fig. 3A). This behavior is characteristic for immunogenic proteins 19 and again more similar to Bet v 1 18 than to Api g 1 which was degraded after 24 h (Fig. 3B). The higher stability of Dau c 1 to endolysosomal processing compared with Api g 1 might be one explanation for its potential allergenicity.

Dau c 1 and Api g 1 also differed significantly regarding cellular cross‐reactivity with Bet v 1. Less than 43% of Dau c 1‐specific TCL and TCC cross‐reacted with the major birch pollen allergen (Tables 1 and 2), whereas 12 of 12 (100%) TCL and 11 of 13 (85%) TCC specific for Api g 1 responded to stimulation with Bet v 1 11. Along these lines, Hofmann et al. 24 had also observed a limited extent of cross‐reactivity between Dau c 1 and Bet v 1. In addition, we found divergent phenotypes of T cells specific for each allergen. The majority of Bet v 1‐cross‐reactive Dau c 1‐specific T cells were Th2/Th0‐like (Table 2). In contrast, most non‐cross‐reactive Dau c 1‐specific T cells belonged to the Th1 subset. We confirmed the more Th1‐like response to Dau c 1 in primary cultures. Sorted T cells proliferating upon a single stimulation with Dau c 1 expressed higher mRNA levels of the Th1 transcription factor Tbet and lower levels of the Th2 transcription factor GATA3 than T cells proliferating upon stimulation with Bet v 1 (Fig. 2). Notably, all Dau c 1‐specific TCC specific for the major T‐cell‐activating region Dau c 1_139–153_ and nonreactive with Bet v 1 showed a Th2‐like phenotype (Table 2). Very recent data demonstrated that cutaneous sensitization of mice to the model allergen papain depended on mast cells and was independent from IL‐33 25. In contrast, inhalant sensitization to papain involved IL‐33 26. Along these lines, we speculate that distinct routes of sensitization involving different pathways, cell types and cytokines may also result in a different distribution of allergen‐specific Th subsets. For example, inhalant sensitization might promote the differentiation of a high proportion of Th2 cells and only a few Th1 cells whereas cutaneous sensitization might promote a more equal but still Th2‐dominated distribution of both subsets. Birth cohort studies provide increasing evidence that sensitization to food allergens happens in early life *via* the skin and gut. These routes of sensitization might also be relevant for Dau c 1 as carrots are frequently consumed by little children who have higher gastric pH values that will impair complete degradation of Bet v 1 and homologous food allergens. Indeed, Bet v 1‐cross‐reactive Dau c 1‐specific TCC displayed a lower expression of the gut‐homing marker integrin β7 and a significantly higher expression of integrin β1 than non‐cross‐reactive clones (Table 3). The latter has been associated with recruitment of T cells to the lung 17. Accordingly, we speculate that Dau c 1 primes T cells in the gut which may not cross‐react with Bet v 1. On the other hand, Bet v 1 primes T cells in the lung which then may cross‐react with Dau c 1.

In summary, we found several indications of a Bet v 1‐independent T‐cell response to Dau c 1 in birch pollen‐allergic patients with carrot allergy. Thus, in addition to cross‐reactivity with the major birch pollen allergen, Dau c 1 itself may show sensitizing activity. This evidence is supported by the demonstration of Bet v 1‐independent IgE responses to Dau c 1 in patients 1, 9, 12, 13 and by immunological properties of Dau c 1 that are characteristic for sensitizing allergens, namely high stability to endolysosomal proteolysis and the existence of major T‐cell‐activating regions. We conclude that Dau c 1 has a special status among Bet v 1‐related food allergens. These insights are relevant for the treatment of carrot allergy and may explain the limited curative effect of birch pollen immunotherapy on birch pollen‐related food allergy.

## Author contributions

N.Z. and B.B. designed the experiments; N.Z., B.N., P.B., A.R., and F.F. performed the experiments and analyzed the data; B.B.W. provided patients' samples, G.J.Z. performed cytokine analysis, and N.Z. and B.B. wrote the manuscript.

## Conflicts of interest

The authors declare that they have no conflicts of interest.
